# Comparing Pixel- and Object-Based Approaches in Effectively Classifying Wetland-Dominated Landscapes

**DOI:** 10.3390/rs10010046

**Published:** 2018

**Authors:** Tedros M. Berhane, Charles R. Lane, Qiusheng Wu, Oleg A. Anenkhonov, Victor V. Chepinoga, Bradley C. Autrey, Hongxing Liu

**Affiliations:** 1 Pegasus Technical Services, Inc., c/o U.S. Environmental Protection Agency, Cincinnati, OH 45219, USA; 2 U.S. Environmental Protection Agency, Office of Research and Development, Cincinnati, OH 45268, USA; 3 Department of Geography, Binghamton University, State University of New York, Binghamton, NY 13902, USA; 4 Institute of General and Experimental Biology SB RAS, 670047 Ulan-Ude, Russia; 5 V.B. Sochava Institute of Geography SB RAS, 664033 Irkutsk, Russia; 6 Irkutsk State University, 664003 Irkutsk, Russia; 7 Department of Geography, University of Cincinnati, Cincinnati, OH 45220, USA

**Keywords:** Lake Baikal, maximum likelihood, near-infrared, Quickbird, random forest, segmentation

## Abstract

Wetland ecosystems straddle both terrestrial and aquatic habitats, performing many ecological functions directly and indirectly benefitting humans. However, global wetland losses are substantial. Satellite remote sensing and classification informs wise wetland management and monitoring. Both pixel- and object-based classification approaches using parametric and non-parametric algorithms may be effectively used in describing wetland structure and habitat, but which approach should one select? We conducted both pixel- and object-based image analyses (OBIA) using parametric (Iterative Self-Organizing Data Analysis Technique, ISODATA, and maximum likelihood, ML) and non-parametric (random forest, RF) approaches in the Barguzin Valley, a large wetland (~500 km^2^) in the Lake Baikal, Russia, drainage basin. Four Quickbird multispectral bands plus various spatial and spectral metrics (e.g., texture, Non-Differentiated Vegetation Index, slope, aspect, etc.) were analyzed using field-based regions of interest sampled to characterize an initial 18 ISODATA-based classes. Parsimoniously using a three-layer stack (Quickbird band 3, water ratio index (WRI), and mean texture) in the analyses resulted in the highest accuracy, 87.9% with pixel-based RF, followed by OBIA RF (segmentation scale 5, 84.6% overall accuracy), followed by pixel-based ML (83.9% overall accuracy). Increasing the predictors from three to five by adding Quickbird bands 2 and 4 decreased the pixel-based overall accuracy while increasing the OBIA RF accuracy to 90.4%. However, McNemar’s chi-square test confirmed no statistically significant difference in overall accuracy among the classifiers (pixel-based ML, RF, or object-based RF) for either the three- or five-layer analyses. Although potentially useful in some circumstances, the OBIA approach requires substantial resources and user input (such as segmentation scale selection—which was found to substantially affect overall accuracy). Hence, we conclude that pixel-based RF approaches are likely satisfactory for classifying wetland-dominated landscapes.

## 1. Introduction

Wetlands are amongst the most productive and biodiverse ecosystems on Earth [1]. However, they have been lost at prodigious rates across the globe [2], and those that remain are imperiled. Junk et al. [3] estimated that 30–90% of global wetlands have been lost, and that climate change and concomitant temperature and sea level rise, along with precipitation pattern changes, will continue to stress the remaining wetlands. Davidson [2] reviewed 189 reports of wetland area changes and determined that 64–71% of wetlands have been globally lost since approximately 1900 AD. With wetland areal loss comes loss in various ecological and environmental functions at both local and landscape scales. For instance, wetlands are known areas of high biogeochemical cycling (e.g., [4,5]), groundwater recharge and stormflow attenuation (e.g., [6,7]), and habitat for many biological species (e.g., [8–10]). Wetland processes that underlie these functions vary by habitat or vegetation structure. For instance, emergent (or non-woody) wetlands perform denitrification at different rates than forested wetlands [11,12]. Similarly, water storage in depressional wetlands—which decouple storm event flows—differs by wetland habitat [13]. Understanding wetland abundance and typology is therefore important to properly managing the existing wetland resources and their concomitant watershed functions.

Satellite remote sensing provides a useful mechanism to delineate, assess, and monitor wetland habitats [14]. Sub-meter to coarse-resolution image data have been analyzed to identify wetlands and demarcate wetland-upland boundaries as well as differentiate habitats within extensive wetland systems (see expansive reviews by [14–16]). Critical decisions on imagery acquisition include resolution, spectral bands, revisit period, and cost. The benefits and detractions of various platforms have been—and will continue to be—assessed and debated as an increasingly large number of satellite systems are launched (e.g., [17]).

Analytical approaches also vary, from visual or manual classification to unsupervised assessments to increasingly complex—and powerful—approaches (e.g., object-oriented classification [18]; random forest [19]; and artificial neural networks [20]). Other approaches include the pixel-based unsupervised Iterative Self-Organizing Data Analysis Technique (ISODATA) [21–24] and the supervised maximum likelihood (ML [24,25]) image classification techniques of change-detection and pattern recognition [24,25].

In contrast with the parametric ML classifier, more recently developed approaches such as random forest (RF) include non-parametric classification algorithms with no assumption of Gaussian distribution of the input/predictor variables. As a powerful remote sensing image classification tool, RF is applicable for both pixel-based or object-based classifications under supervised or unsupervised settings [19,26,27]. Compared to ISODATA and ML, RF also has an advantage in providing the relative importance of the input variables in predicting the response variable by permuting the predictor variable value and measuring the error estimate before and after the permutation [26,28,29].

Unlike the aforementioned unsupervised and supervised pixel-based techniques (e.g., ISODATA and ML), the object-based image analysis (OBIA) approach considers contextual spatial information such as shape, smoothness, and compactness of geographical features of interest at different spatial scales [30,31]. However, OBIA workflow for image classification involves an iterative trial-and-error image segmentation and optimization step. This is followed by a bottom-up merging of image-objects with the spatial and spectral heterogeneity threshold of adjacent landscape objects constrained by a user-defined scale parameter, with a subsequent step of classifying the primitive image-objects at the object-level using training data [30].

Numerous studies have used both pixel- and object-based image classification techniques with RF for various natural resource management applications, including wetlands. For instance, Husson et al. [32] used 5-cm spatial resolution true-color unmanned aircraft systems data for mapping non-submerged aquatic vegetation, classifying water (vs. vegetation), growth form, and dominant taxon using OBIA and RF classifiers, with overall accuracy results obtained for RF ranging from 62–90% for the growth form type to 52–75% for the dominant taxon classifications. Mahdianpari et al. [33] used synthetic aperture radar (SAR) data in a hierarchical object-based RF approach to discriminate eight herbaceous wetland cover types in the Canadian province of Newfoundland with an overall accuracy of 94% achieved. Dronova et al. [31] used the 32-m Beijing-1 satellite data and fuzzy supervised classification methods and an OBIA technique to detect changes of the major wetland cover types (i.e., water, mudflat, vegetation, and sand) of Poyang Lake, the largest freshwater lake–wetland system in China, with comparatively higher overall accuracy achieved for vegetation and water (90% and 82%, respectively). Ariel et al. [34] applied a set of spatial and spectral image-object metrics to classify water and four vegetation types using RF with an overall accuracy of 92%. Tian et al. [35] found higher overall accuracy using the OBIA coupled with the RF classifier (overall accuracy 93%) when compared to support vector machine and artificial neural network approaches in classifying nine land cover types, including wetlands.

Pixel- and object-based classification of landscape components is frequently further improved through the inclusion of spatial and spectral metrics in the algorithms. That is, in addition to the direct use of the spectral bands of the chosen sensor, numerical band combinations and band ratios may provide additional information [31]. For instance, the well-known Normalized Difference Vegetation Index (NDVI [36]) can be used as a proxy variable for indicating the presence and condition of vegetation (e.g., vigor, health, and abundance). This index can vary by vegetation types and habitats, thus providing useful information for improving land cover classification. Other vegetation indices have improved classification of remotely sensed data; their use is frequently dependent on site-specific conditions. For instance, to minimize the atmospheric aerosol scattering effect, the atmospherically resistant vegetation index (ARVI) [37] has been derived. Similarly, soil brightness can affect vegetation indexes, and this can be compensated by using the soil-adjusted vegetation index (SAVI [38]). In another example, to simplify or reduce computation time and computer processing power requirements, the infrared percentage vegetation index (IPVI) has been used to replace the NDVI [39].

Furthermore, auxiliary input variables such as digital elevation model (DEM) and spatial metrics such as derivatives of the Grey Level Co-occurrence Matrix (GLCM) are also extensively used in various studies for improving land cover classification and prediction accuracies [28,31,40]. Topographic position, through its effect on hydrological processes [41] such as the prediction of areas of soil saturation in low-lying areas [31], can influence the distribution of wetland classes in a landscape. Rodriguez-Galiano et al. [40] have used elevation, slope, and aspect variables derived from a digital terrain model along with Landsat 5 TM spectral data as input to a RF model to classify 14 land cover categories in Spain with overall accuracy of 92% [40]. Wright and Gallant [42] have found DEM-derived topographic variables to be relevant in improving wetland mapping by increasing their accuracy in better identifying and differentiating upland areas from wetlands.

These varied random forest and object-based classification approaches and different metrics are useful in assessing and classifying landscape components. However, the methods discussed above also suffer from limitations. It is difficult and resource-intensive to collect sufficiently large amounts of field data for training an object-based (RF) model. Despite widely reported improved performance of object-based over pixel-based image classification approaches, Dronova [31] indicated results vary by data type, spatial scale, and research objectives when applied in complex wetland systems (e.g., large wetlands with varied wetland vegetation structure and open water).

Wetlands in particular can be challenging landscape elements to classify due to their ecotonal location at the terrestrial–aquatic interface and complex hydroperiod and hydro-patterning which control the vegetation structures found therein. However, their importance in contributing to landscape hydrological, biogeochemical, and habitat functions and massive wetland losses worldwide [2,3] make assessing the location and structure of wetland systems a critical research need. With the beguiling and varied approaches to classifying wetland systems, in this study we sought to determine if pixel-based or objected-based approaches performed most satisfactorily in an analysis and classification of the lower Barguzin River Valley, a large wetland study area draining into Lake Baikal, a United Nations Educational, Scientific, and Cultural (“UNESCO”) World Heritage Site located in Siberian Russia. We furthermore compared the efficacy of parametric (ISODATA, ML) and non-parametric (RF) approaches and iteratively analyzed outputs with spatial and spectral classification metrics seeking a parsimonious and effective wetland classification solution. Thus, in addition to accurately classifying the wetland landscape to improve management options, we aimed to provide an assessment and recommendation of methodological approaches for consideration specific to classifying wetland landscapes.

In our literature review, we did not find wetland classification studies assessing and applying highly numerous structural or habitat classes. For instance, an extensive review of OBIA for wetland mapping [31] indicated few classified wetland systems into more than 10 or 11 classes (e.g., open water, emergent marsh, submergent vegetation, etc.). Our review suggested that overall accuracy in wetland studies frequently fell below an arbitrary benchmark of ~85% when the total number of wetland-specific classes exceeded four.

Thus, in our field-based analyses of a large wetland system in Siberian Russia with nearly 20 different structural and vegetative habitat and wetland classes, we sought analyze the differences in overall accuracy when comparing between three classification methods (pixel-based ML and RF, and object-based RF), constraining our analyses to use the same input field and remote-sensing datasets. The outcome of this study, therefore, assists end-users in selecting (and parameterizing) the proper classifier to analyze the structure of the world’s imperiled and complex wetlands.

## 2. Materials and Methods

### 2.1. Study Area

**Figure 1:**
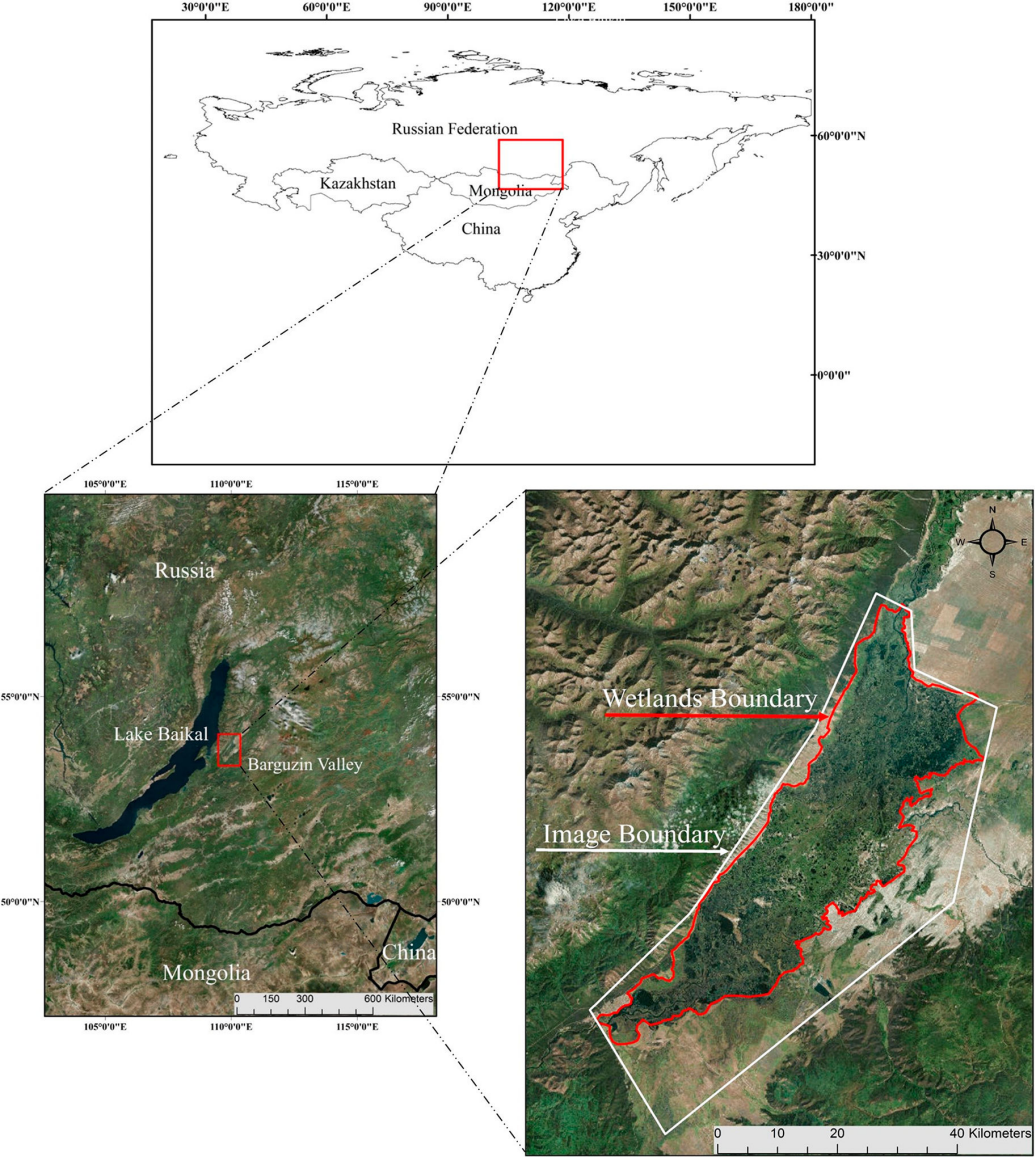
Location of Lake Baikal and the lower Barguzin Valley study area and Quickbird imagery boundary.

The Barguzin River is a major tributary to Lake Baikal, the oldest, deepest (~1600 m), and most voluminous freshwater lake in the world with a catchment area of approximately 571,000 km^2^ [43–45]. Located on the eastern boundary of Lake Baikal in south-central Siberia, Russia, the Barguzin River flows approximately 480 km with an average slope of 2.8% before reaching Lake Baikal, where it provides ~9% of the total inflow to the lake [46,47]. The extensive wetland area in this study (approximately 500 km^2^, Figure 1) is termed the lower Barguzin Valley. The area experiences prolonged seasonal flooding associated with valley narrowing as the river reaches the southern edge of the Barguzin Mountain Range on the eastern Baikal periphery [46]. The regional climate is continental, with long, cold, and relatively dry winters [46]. Land use in the region is mostly undeveloped, with pastoral and subsistence farming. Both mining and forestry activities also occur in the approximately 21,000 km^2^ watershed.

### 2.2. Image Acquisition and Processing

**Figure 2:**
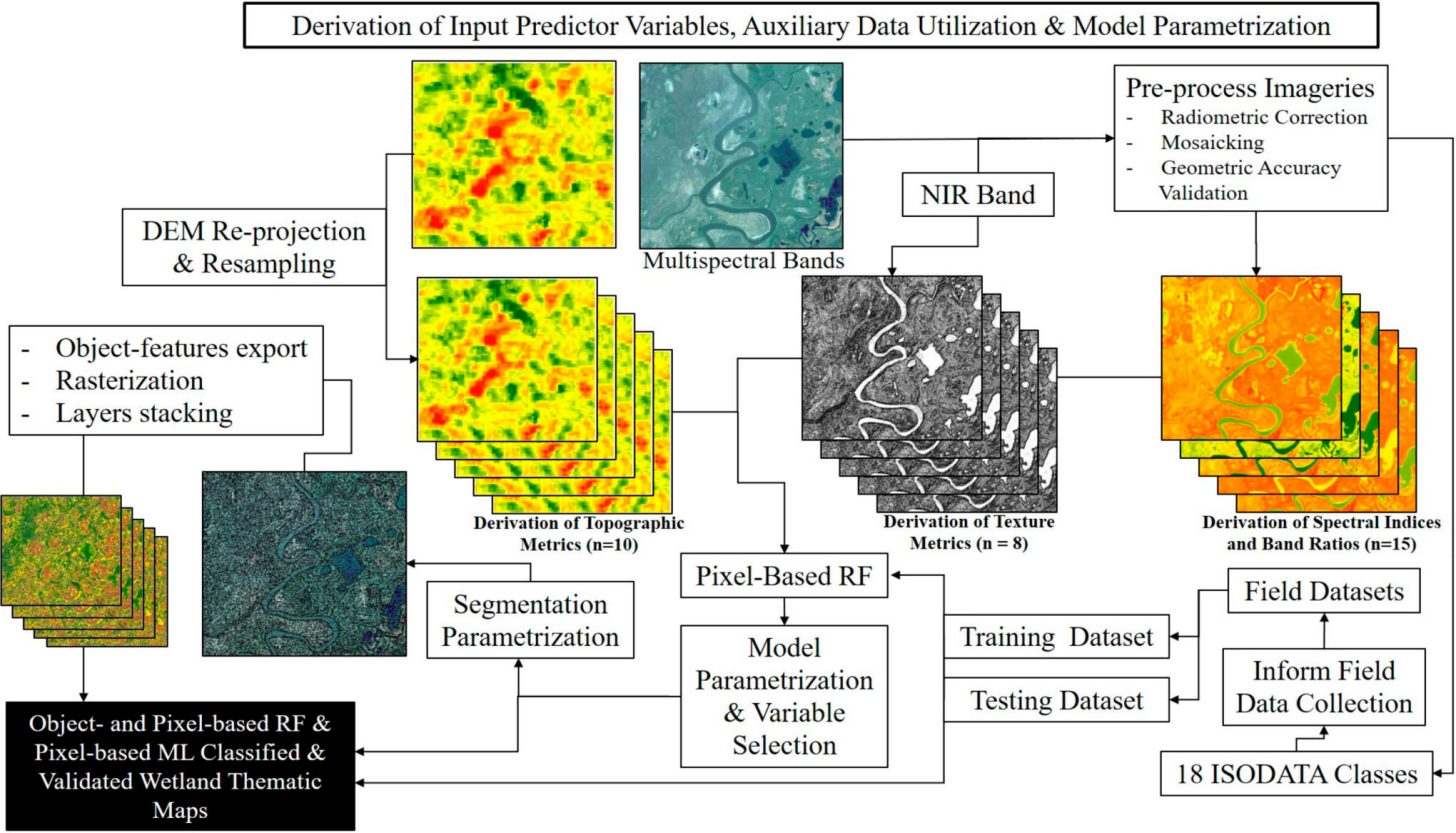
Field and remote sensing data-processing workflow.

We acquired ortho-ready Quickbird imagery (DigitalGlobe, Westminster, CO, USA) for four cloud-free dates in 2012: 7 June, 16 June, 22 June and 12 August with mean off-nadir view angle of 20.8°, 17.8°, 26.4° and 17.0°, respectively. The Quickbird satellite collected panchromatic data (0.6-m nominal pixel size) plus four multispectral bands (at 2.4-m nominal pixel size): blue (B1, 450–520 nm), green (B2, 520–600 nm), red (B3, 630–690 nm), and near-infrared (B4, 760–900 nm). Nominal locational accuracy was 23 m at nadir. The data-processing and workflow diagram is given in Figure 2. The images were converted to top-of-the-atmosphere (TOA) reflectance and mosaicked in PCI Geomatica (PCI Geomatics Enterprise, Inc., Markham, ON, Canada), taking advantage of the 0.2–0.8 km wide overlap between image tiles.

We conducted an initial unsupervised classification of the study area using the mosaicked four-band Quickbird TOA reflectance data (bands 1–4) and the aforementioned Iterative Self-Organizing Data Analysis Technique clustering algorithm (ISODATA; [21,22,48]) in ENVI (Harris Geospatial Solutions, Herndon, VA, USA, version 5.3). We arbitrarily began with 40 spectrally based thematic classes. We reduced these to 18 reasonably distinct classes for field analyses; 95% of all pairwise comparisons between unsupervised classes had Jeffries–Matusita separability values ≥1.8, indicating effective class fidelity [12,49,50]).

### 2.3. Field Data Collection

**Figure 3:**
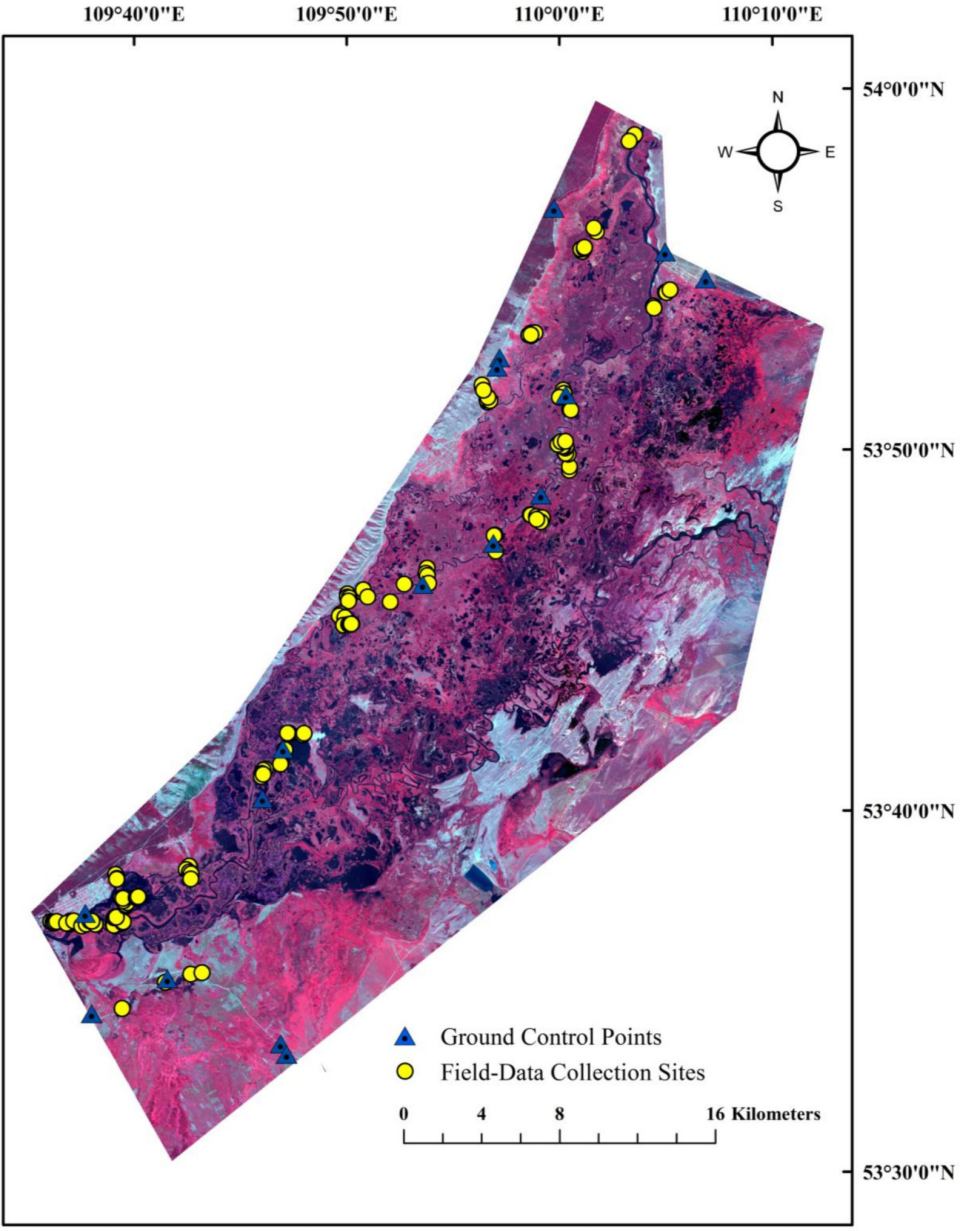
Field survey (*n* = 142) and ground-control sites (*n* = 16) within the lower Barguzin Valley study area overlain on an image composite (Quickbird bands 2, 3, and 4).

We collected field data from 6–10 unique locations in the Barguzin Valley for each of the 18 initial ISODATA classes during an expedition in late August 2013. Sites were selected based on accessibility to roads, along river courses (for access via boats), and through a combination of approaches. Following Lane et al. [12,50], two teams of ecologists and botanists collected vegetation and habitat data from 142 vegetation plots (Figure 3). Data collected at each 100-m^2^ plot included dominant species (≥5% cover) and water depth. Photographs were taken from the center outwards at all cardinal points, as well as straight down. If more than 5% of the plot was determined to be bare ground, open water (without ≥5% vegetation), filamentous algae, or thatch from graminoids (e.g., *Carex* spp.), that information was noted and used in the classification analyses below. We located the approximate center of each plot by averaging 20 GPS location readings of either a Trimble Nomad or a Trimble Yuma (Sunnyvale, CA, USA), with 2–5-m real-time accuracy. The collected species-level vegetation data were subsequently combined to genus-level relative abundance data for use in this study. Sixteen ground control points (GCPs) were collected and used to validate the geometric accuracy of the images. Non-ambiguous, man-made physical structures and road intersections were used for this purpose (see Figure 3); at each GCP, 100 GPS readings were averaged using the GPS receivers noted above, and photographs were taken of the GCP sites. Quickbird multispectral data were pan-sharpened using the Gram-Schmidt Pan-Sharpening method in ENVI for GCP validation. All but two GCPs were within two Quickbird-pixels (i.e., within 1.2 m) from their true ground location. Due to significantly high computational power and processing time requirements, the original non-pan-sharpened multispectral bands were used for classification.

### 2.4. Spatial/Spectral Metrics and Geospatial Data

We calculated 37 input predictor variables for our supervised classification of the Barguzin Valley study area (Table 1). Although many are similar, there are useful differences amongst the indices which we anticipated could be used to improve the fidelity and specificity of our class discrimination. We conducted univariate linear correlation analyses amongst the variables (Table 2) that informed our subsequent analytical approaches. The variables include (see Table 1): Normalized Difference Vegetation Index (NDVI; [51]), Blue Normalized Vegetation Index (BNDVI; [52]), Green Normalized Vegetation Index (GNDVI; [52]), Atmospherically Resistant Vegetation Index (ARVI; [53]), Difference Vegetation Index (DVI; [51]), Soil Adjusted Vegetation Index (SAVI; [38]), the Infrared Percentage Vegetation Index (IPVI; [39]), Normalized Difference Water Index (NDWI; [54]), Water Ratio Index (WRI; [55]), and various Band Ratios (i.e., Blue/Green, B1/B2; NIR/Blue, B4/B1; NIR/Green, B4/B2; NIR/Red, B4/B3; Red/Blue, B3/B1; and Red/Green, B3/B2; [52]). Although certainly not a complete list of metrics, these vegetation indices and band ratios were selected to assist in discriminating amongst similar wetland habitats typically differentiated by botanists and ecologists based on vegetation structure. We additionally included eight spatial metrics of texture (Contrast, Correlation, Dissimilarity, Entropy, Homogeneity, Mean, Second Moment and Variance) calculated using the QuickBird NIR band, the native DEM value, and an additional 10 topographic metrics (Aspect, Cross-sectional Convexity, Longitudinal Convexity, Maximum Curvature, Minimum Curvature, Plain Convexity, Profile Convexity, RMS Error and Percent Slope) calculated using DEM data to further differentiate among vegetation and habitat classes in the study area [56]. DEM-based metrics were derived using elevation data from the Advanced Spaceborne Thermal Emission and Reflection Radiometer-Global Digital Elevation Model (ASTER GDEM). For processing compatibility with the rest of the input predictor variables, the ASTER GDEM (nominal pixel size: 24.2 m) was re-projected to WGS-1984 UTM-Zone-49N and resampled to the original Quickbird multispectral nominal pixel size of 2.4 m.

### 2.5. Supervised Image Classification

Subsequent to the field expedition, we developed supervised classification maps of wetland vegetation using both pixel-based and object-based approaches. As random forest is sensitive to uneven class distribution [27,28], while we initially conducted our analyses with unbalanced datasets, we report here the results using a balanced approach (*n* = 125). Approximately 70% of the points were randomly selected as the training set (*n* = 89), and the rest as the validation set (*n* = 36), with an equal number of data points allocated for each class (i.e., five and two points per class for training and testing, respectively). The one exception is for Class 10, which only had four points available for training. The partition into the training and testing datasets was performed by visually inspecting the point distribution so that the training and/or the testing datasets were distributed in space to maximize distances between points thereby minimizing the chances of spatial autocorrelation. We then delimited regions of interest (ROIs) for each of the 18 thematic classes from the 125 field survey sites used in the balanced approach. Each ROI was approximately 30 pixels in area and most, though not all, consisted of a single thematic class. In certain cases, the ROIs—which otherwise were centered around the field-based data point—were moved slightly to maintain thematic homogeneity while staying within the approximate bounds of the field-based sampling area.

#### 2.5.1. Pixel-Based Random Forest Classification

The random forest (RF) classification algorithm was implemented in R (RStudio, Inc., Boston, MA, USA, version 0.99.90) using the package randomForest [57]. Two-thirds of the ROI training data described above were used for RF classifier training and the remainder were “out-of-bag” (OOB) samples for estimating internal classification error. Exploratory analyses determined that the highest predictive power was found with the following settings: two variables (mtry), 1000-tree maximum (ntree), and 1000 bootstrap (or OOB) samples in the classification; these values were used in the final model run. The accuracy of the pixel-based RF model was assessed with pixels from the 30% hold-out ROIs across the study area. RF was conducted on the combination of four data layers (Quickbird bands 1–4) as well as bands 1–4 plus 33 additional input variables described above (see Table 1). Random forest usefully provides quantitative variable importance measures, the Mean Decrease in Gini (MDG) and the Mean Decrease in Accuracy (MDA) values. MDG informs the accuracy of a particular class due to utilization of a given variable while the MDA provides the difference between OOB error of the original dataset and the OOB error from random permutations of a set of input predictor variable values [26].

#### 2.5.2. Pixel-Based Maximum Likelihood Classification

Subsequent to our RF-based approach, we conducted the Maximum Likelihood (ML; [58,59]) supervised method to classify the ROI training data into 18 supervised classes using the predictor variables that yielded the highest overall accuracy with the most parsimonious pixel-based-RF model (i.e., Quickbird band 3, WRI, and mean texture computed from Quickbird band 4; see results further described below). These layers were chosen because preliminary results using the pixel-based-RF model demonstrated only slight changes in accuracy (both positively and negatively affecting our results) with additional metrics. Furthermore, as many spectral metrics were highly correlated (e.g., |r| values ≥ 0.89; see Table 2), we sought to develop a parsimonious model that balanced processing requirements and accuracy and hence we initially limited the analyses to a three-layer stack. However, recognizing that additional bands may potentially improve the overall ML accuracy, two additional variables that comprised the five most important variables as determined by MDG, (i.e., Quickbird bands 2 and 4, see below) were also analyzed along with the aforementioned three predictors.

#### 2.5.3. Object-Oriented Random Forest Classification

Contrary to human landscapes with relatively well-defined geometric structures, natural environments such as wetlands have fewer “objects” with discrete distinctive spatial shapes [28,31]. That is, the natural environment tends to grade from one ecotype to another. However, as patterns emerge within and between natural systems, we explored the utility of an object-based image analyses (OBIA) classification of the study area using multi-resolution image segmentation in eCognition Developer (Trimble, Inc., Munich, Germany, v. 9.2). OBIA in eCognition is a hierarchical, region-growing segmentation algorithm where objects of similar properties starting from a single pixel are merged until the weighted intra-object heterogeneity is smaller than a defined scale parameter.

**Figure 4:**
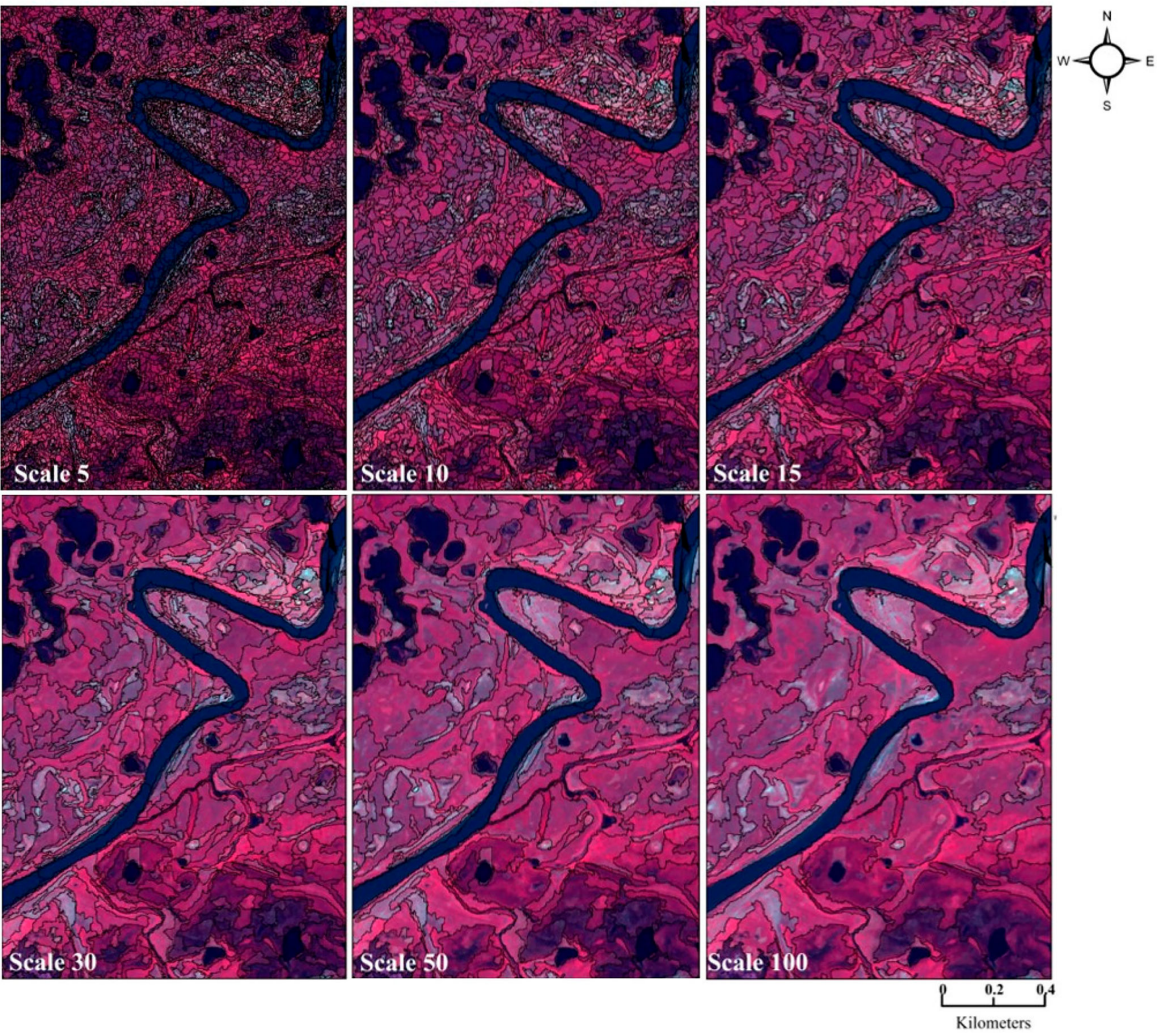
Example of image-objects created at different segmentation scales using parameters of shape = 0.1 and compactness = 0.5. The number of objects created across the study area decreased with increasing segmentation scales: 5 (5,191,948 objects), 10 (1,474,823 objects), 15 (711,351 objects), 30 (204,026 objects), 50 (81,026 objects), and 100 (23,091 objects).

Segmentation scale affects the object size and hence the properties of discrete objects in the study area. We analyzed multiple scales in turn (e.g., 5–100; Figure 4) and determined via accuracy assessment that segmentation at the spatial scale of 5 provided the optimum image-object size. Color (multispectral heterogeneity) and shape (a function of smoothness and compactness heterogeneity) are the primary image-object features that are used for image-segmentation and for improvement in primitive image-object creation [30]. We segmented color (i.e., spectral band information) and shape (compactness and smoothness metrics) to generate optimum pixel groups (i.e., image-objects) exhibiting intra- and inter-object spectral homogeneity and heterogeneity [60]. Initial analyses of the study area informed our decision to use shape and color segmentation weights of 0.1 and 0.9, and compactness and smoothness parameters of 0.5, respectively. Upon segmentation completion, we calculated the spatial and spectral metric average values for each of the 5,191,948 segmented objects in the study area. We then utilized the RF approach described above to classify the study area. Similar to the pixel-based ML approach, OBIA-RF was performed using three- and five-stack layers to provide the opportunity to contrast amongst the methods in wetland classification.

### 2.6. Classification Accuracy

We assessed omission and commission errors of the 18 delineated and smoothed [61] classes using a 70:30 approach wherein 70% of the ROIs were randomly selected for training and the remaining 30% were used for validation of the pixel-based ML and RF and OBIA-RF approaches described above. We also conducted and report overall accuracy [62]. We quantitatively assessed if the observed difference in the classification accuracies between the three classifiers were statistically meaningful using McNemar’s test [63,64].

## 3. Results

### 3.1. Field Data Collection

Fifty-six different genera were identified as occurring at >5% frequency across the sampling locations. Members of the genera *Carex* and *Equisetum* were more commonly found (46 and 25 times, respectively), followed by *Calamagrostis, Myriophyllum, Agrostis, Nymphoides*, and *Potentilla*. Twenty-three genera were only encountered a single time. We also noted the >5% abundance of open water (49 sites), thatch (33 sites), and bare ground (22 sites).

### 3.2. Classification Approaches

Satisfactory utilization of high-resolution geospatial products to detect and delineate wetland classes and aquatic habitats was achieved with the marriage of multispectral satellite and field data. Varied results were found using either pixel- or object-based approaches and a parsimonious suite of spatial and spectral metrics, which appeared to have sufficient class specificity and fidelity for successful classification. Accuracy was assessed through analyzing the ROIs from the hold-out dataset (*n* = 36). We developed and reported a final classification based on each approach and report both a class-based confusion table and both producer’s and user’s accuracy for each class (by classification type). We did not delve into the synecological vegetative or descriptive characteristics of each of the 18 thematic classes in this manuscript though these analyses are currently underway (see, e.g., [12,50]). A comparison between classification approaches using McNemar’s test resulted in no significant difference observed among the three classification methods (Table 3).

#### 3.2.1. Pixel-Based Random Forest and Maximum Likelihood Classification

**Figure 5:**
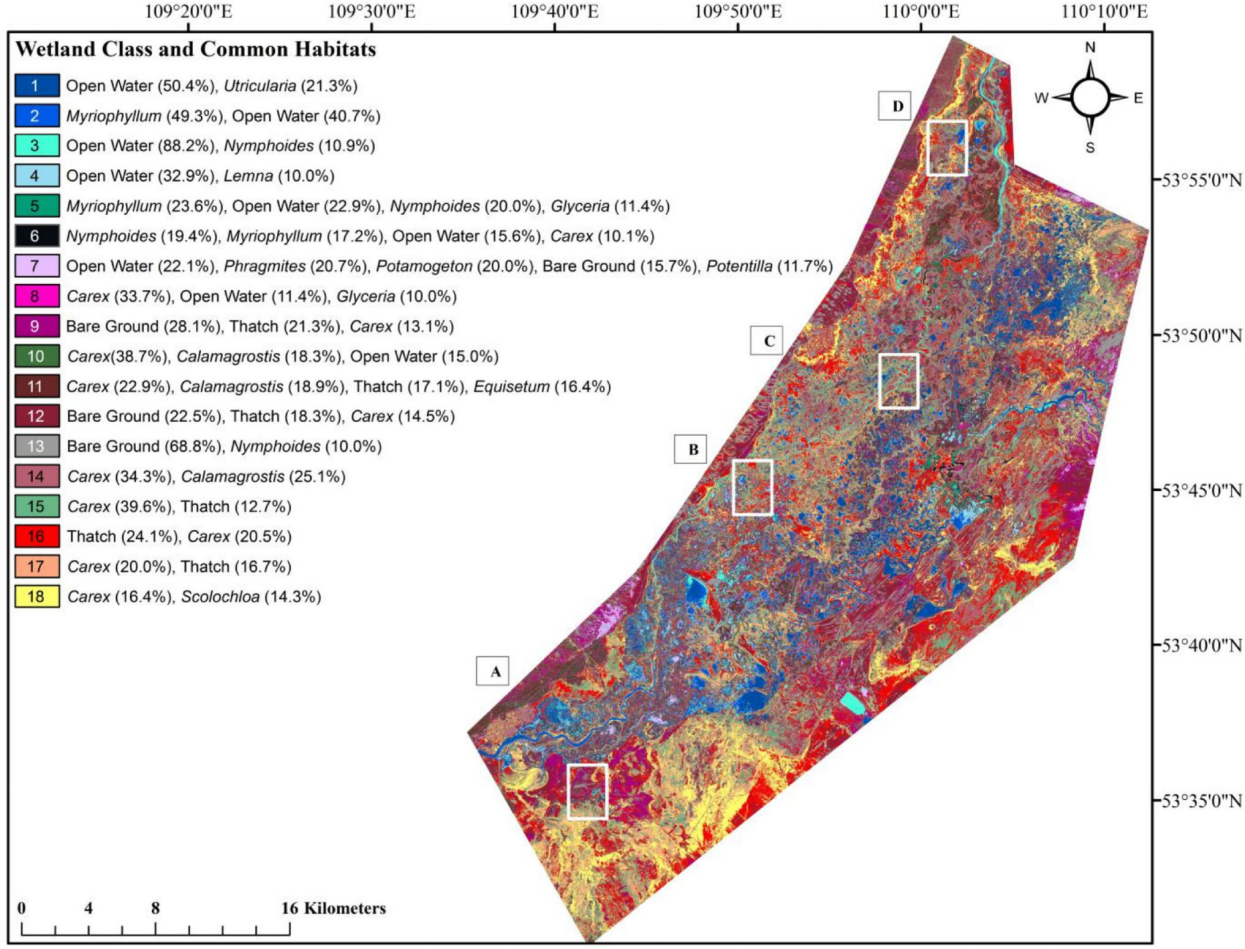

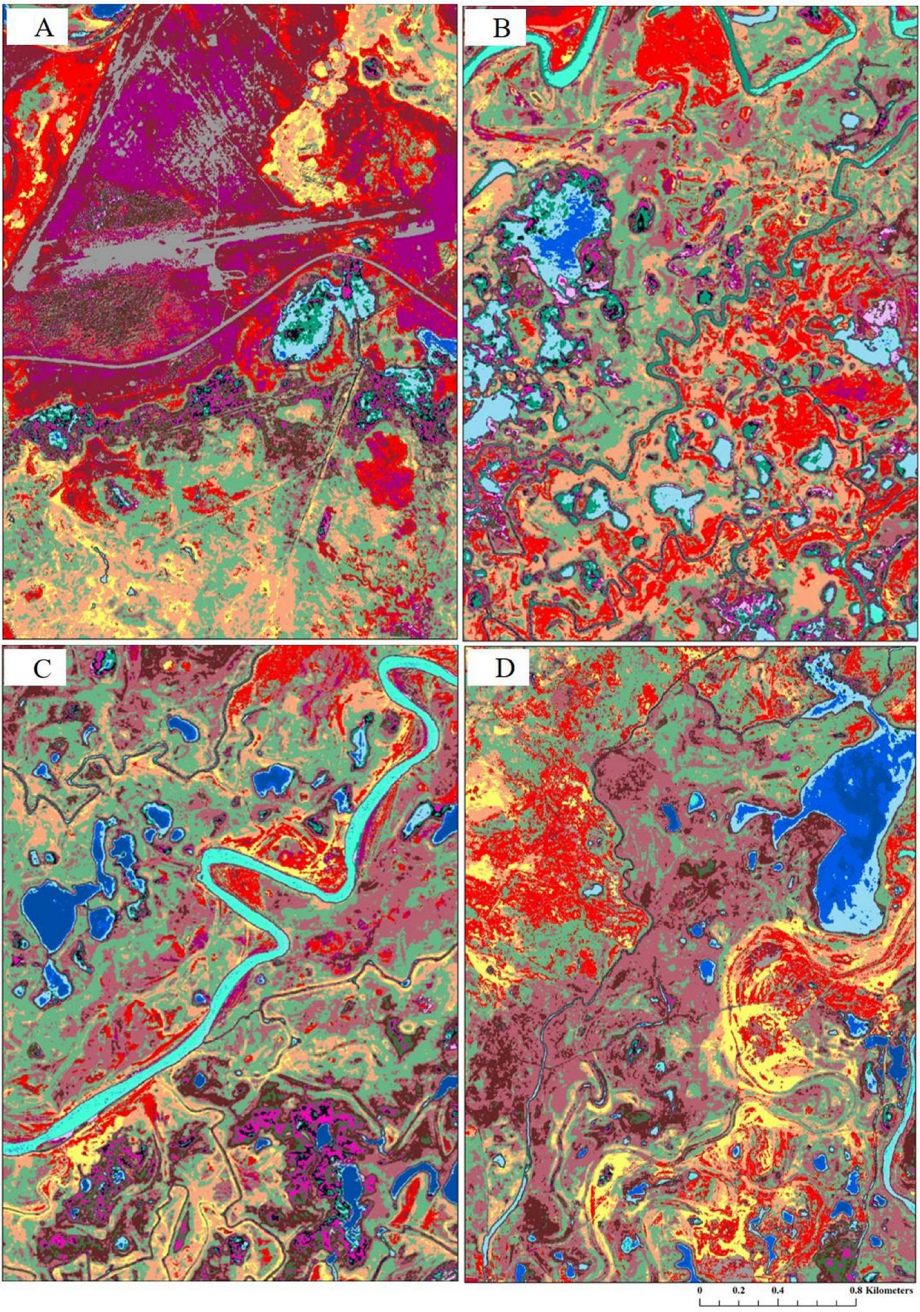
(**a**) Genus-level wetland and aquatic habitats classification map (pixel-based RF approach using three-layer stack). Four focal areas (5**A**–5**D**) are shown in finer detail in (**b**). Percent values given in parentheses represent the approximate abundance of each genus or habitat found in the field-based analyses for each class. (**b**) Finer-detailed genus-level wetland and aquatic habitat classification thematic maps developed using the pixel-based RF approach for the areas of interest shown by white-colored squares in (**a**). Classes correspond to the legend in (**a**).

Pixel-based classification was conducted using both the RF and ML approaches. The pixel-based RF approach was conducted with various predictor combinations, with overall accuracy ranging from 72.7% to 87.9% (Table 4). The highest overall accuracy, 87.9%, for pixel-based RF classification was achieved using Quickbird band 3, WRI, and mean texture (Figure 5a,b and Tables 4 and 5). This highest overall accuracy was achieved using the aforementioned three variables despite the fact that WRI and mean texture are highly correlated (|r| = 0.99; see Table 2). The overall accuracy, however, decreased to 80.1% when the 22 non-correlated variables were considered with the exclusion of WRI and inclusion of mean texture; accuracy dropped to 72.7% when mean texture was excluded (see Table 4). These results indicated it is paramount to include both mean texture and WRI for meaningful improvement in prediction accuracy of the wetland classifications—although mean texture without WRI improved the overall accuracy (e.g., from 72.7% to 80.1%).

A benefit of the RF approach is the derivation of variable importance factors based on the MDG values. We assessed MDG based on 100 RF runs. For the pixel-based RF, Quickbird bands 2, 3, and 4, WRI, and mean texture were consistently ranked as the five most important variables, the rank of importance for the remaining variables changed with different RF runs of the 100 iterations (Table 6). As the analytical process for ML and OBIA can be laborious, we used the MDG to inform our selection of variables to use in the ML and OBIA approaches.

The ML approach was conducted using the same three-layer stack image as the RF approach, and resulted in an overall accuracy of 83.9% (Table 7). ML analysis using the five most important predictor variables from the pixel-based RF analysis (Quickbird band 2–4, WRI, and mean texture) resulted in a decrease in overall accuracy from 83.9% to 80.2%.

Perfect producer’s (PA) or user’s accuracy was calculated for the pixel-based RF and ML approach class confusion matrix (Tables 5 and 7) using three bands for 11 and 8 of the 18 classes, respectively. Comparatively higher classification error using pixel-based RF approach occurred with Class 3 (66.9% error), Class 6 (34.5% error), and Class 14 (35.6% error). Greater PA errors in the ML analyses occurred with Class 3 (30.2% error), Class 5 (33.3% error), Class 6 (52.5% error), Class 9 (61.9% error), and Class 14 (48.4% error). The errors suggested open water, emergent, and submerged vegetation were more difficult to classify with remotely sensed data.

#### 3.2.2. Object-Oriented Classification

The OBIA classification was conducted on the highest-performing three- and five-layer stacks of the pixel-based RF approaches, consisting of Quickbird band 3, WRI, and mean texture for the three-layer stack and those plus bands 2 and 4 for the five-layer stack. We did not analyze additional permutations due to the high OBIA computational and resource requirements of analyzing the full suite of spatial and spectral metrics (see, e.g., Table 1) and then iteratively conducting RF on the over five million objects in the study area. We explored the effects of scale on our results using three layers. The highest three-layer OBIA accuracy rate (84.6%) was achieved using a segmentation scale of 5. Increasing the scale typically resulted in decreasing overall accuracy with the poorest results at the scale of 100 (37.4%; see Table 8). The only exception to this is at segmentation scale of 50 where the overall accuracy was higher than at segmentation scale of 30.

In addition, we saw an improvement in overall accuracy from 84.6% (with three layers and OBIA) to 90.4% (with five layers and OBIA; Table 8). The likelihood of diminishing returns with additional data layers (and concomitantly the substantial resource requirements) through this approach informed our decision to use no more than five layers in our OBIA.

## 4. Discussion

### 4.1. Random Forest in Wetland Classification

Targeting overall accuracy above an arbitrary benchmark acceptable value of 85% in classifying complex wetland systems can be challenging [31], a challenge exacerbated by the inclusion of numerous wetland classes (i.e., 18 in this study). Studies using high-resolution satellite data may consider the utility of “high-resolution” ecological data (i.e., field-based community data that go beyond structural classifications) in their systems of interest—although we hasten to add that the level of specificity is dependent on the questions asked and management purpose for which the data are often requested. High accuracy is also dependent on abundant field-based data, as well as cloud-free imagery and a combination of ecologically and spectrally concordant classes.

**Figure 6:**
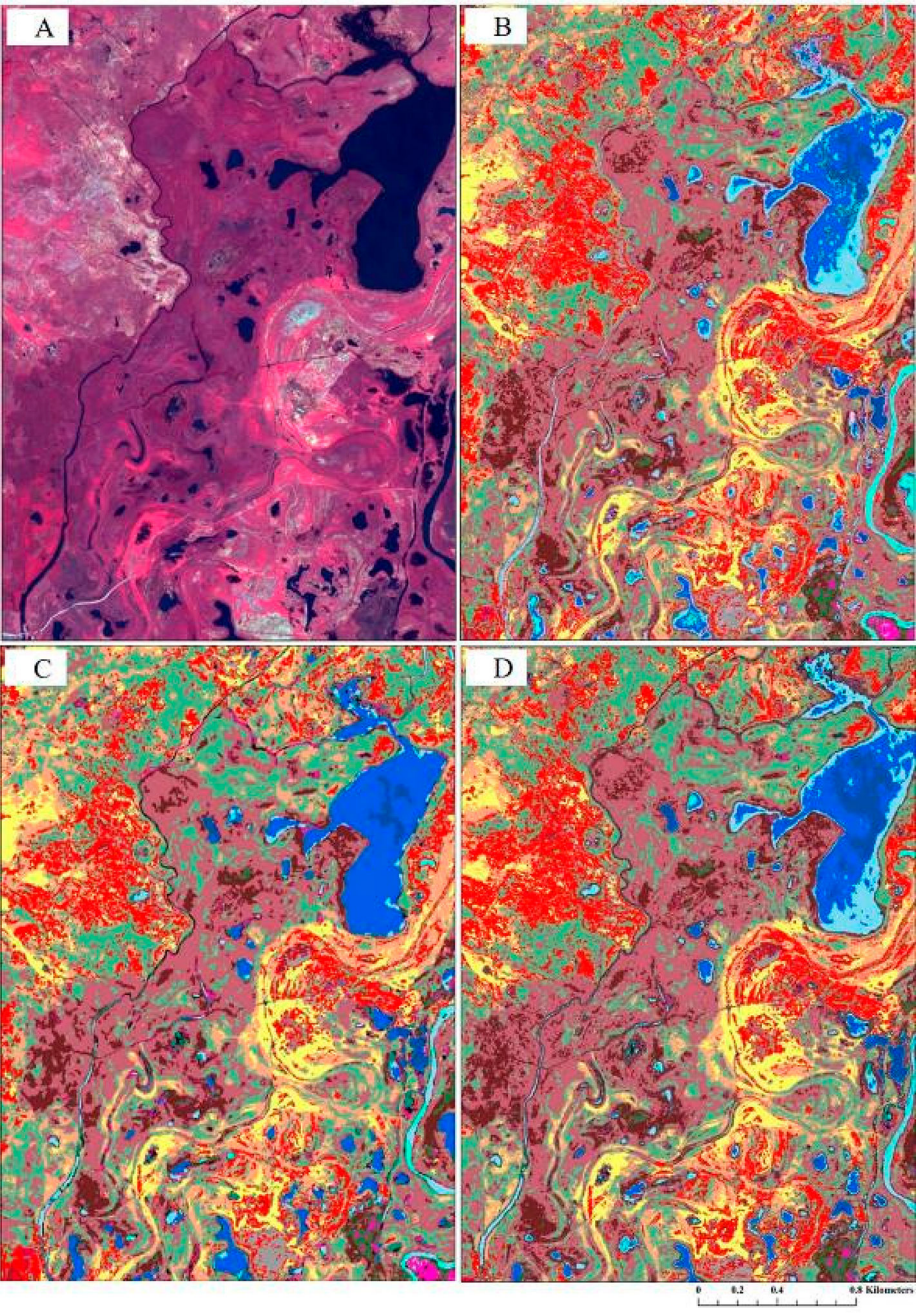
Contrasting the results between the varied methods using a three-layer predictor dataset for inset-5D in Figure 5a: (**A**) Quickbird imagery color composite of bands 2, 3, and 4; (**B**) pixel-based maximum likelihood classification; (**C**) object-based random forest classification; and (**D**) pixel-based random forest classification. Classes correspond to the legend in Figure 5a.

Accuracy is furthermore a product of the analytical approach employed and the number of predictor variables. In this study, we found the greatest overall accuracy to be achieved with the OBIA and RF classifier (~90%) using five predictor variables (and segmentation scale 5); this decreased to <85% when three predictors were used. Pixel-based RF achieved 88% overall accuracy using only three predictors, and, as Duro et al. [65] also reported, required substantially less user interaction and processing time than conducting the OBIA. Furthermore, though differences are evident when visually comparing between the approaches (e.g., Figure 6), McNemar’s test found no significant overall difference between the varied approaches (see Table 5). Thus, although any of the approaches may be employed, our findings suggest a pixel-based RF approach may be best suited when considering classifying the wetland-dominated landscape.

That random forest has many advantageous features to consider in remote sensing applications is becoming increasingly evident [29,40,57,66]. Some of the advantages of RF include the fact that it is freely available (e.g., in the R statistical package) and relatively user-friendly with limited user selections required (in our instance, the number of variables for each node in a random subset of data (mtry) and the number of trees to grow in a forest (ntree) were specified). RF is computationally efficient in handling large datasets, even those with substantial “noise” and the presence of outliers [29,40]. The fact that RF uses the input-predictor variables with replacement but not deletion (i.e., bagging) apparently allows more specificity when classifying, and as a non-parametric approach there are fewer restrictions on the distribution of the input data layers. Lastly, RF output includes the generation of variable importance (MDG) and unbiased internal estimates of error (MDA), which assists in the interpretation of the results [40,57]. Limitations of RF include the “black box” nature of the model, where limited information about the relationships between the predictor variables and the response variables can be discerned [66]. Furthermore, RF results change when an unbalanced study design is employed [27,28]. We endeavored to sample at least five sites, and as many sites as ultimately possible for each class, during our short sampling window for the field-based expedition. However, following the suggestions (i.e., [27,28]), we ultimately did not use some of the data points acquired during the sampling period, which resulted in lost sampling efficiency and resource use.

However, the benefits of RF appear to outweigh the limitations, and consequently, numerous studies have used RF as the classifier of choice for both pixel- and object-based image classifications for various applications using data from multiple remote sensing platforms. For instance, Mallinis et al. [67] conducted a RF analysis using Quickbird to delineate vegetation polygons of dominant species for national forest database creation, and Smith [68] classified land cover classes using SPOT. The literature is increasingly replete with the use of RF in classifying landscapes (e.g., [19,25,69–71]).

### 4.2. Variable Importance in Wetland Classification

As noted, the use of RF makes a quantitative analysis of variable importance possible through MDG (Mean Decrease in Gini). For the pixel-based RF, Quickbird bands 2, 3, and 4, WRI and mean texture were consistently ranked as the five most important variables (see Table 6). It is not surprising that Quickbird band 4, mean texture (derived from band 4), and WRI were the three most important variables in class discrimination. As water column absorption of energy in the NIR spectrum (i.e., band 4) is high [72], the abundance of water, a factor controlling wetland vegetation community development, would feature prominently in categorizing the studied system. Similarly, photosynthetically active vegetation reflects more energy in the near-infrared portion (e.g., [73]) while absorbing more energy in the blue and in the red-light regions of the spectrum. The WRI assess the abundance of the green, red and near-infrared band values to the blue band; with abundant water associated approximately with lower (<2.5), barren land with intermediate (2.5–4.0) and vegetated areas with higher (>4.0) WRI values. The Barguzin Valley is dominated by an extensive and spatially connected surface water hydrological system (submergent marshes, rivers, lakes, etc.) and the role of WRI is in differentiating the valley’s aquatic habitats from the uplands. This is particularly true in differentiating mixed pixels belonging to the water-soil and water-vegetation traditional zones. As wetland vegetation was the main study object in this analysis, differences in spectral reflectance (Quickbird bands 2–3) would logically follow as a second group of important variables in group determination. It was, however, somewhat surprising that the abundant additional metrics that we calculated in an effort to provide greater discernment between wetland classes were not particularly relevant to the classification, as evidenced by their lack of relative importance and the decrease in overall accuracy when additional metrics were included (for the pixel-based approaches). Both the RF and ML approaches decreased in overall accuracy with increased predictors beyond our three-stack parsimonious model.

One potential reason for the lack of specificity with increased metrics is simply a limitation of the input data set. Quickbird has limited spectral bands (only four plus the panchromatic band), thus limiting to a degree the possible permutations of the data, at least the data based on Quickbird’s available spectra (limited vis-à-vis more current satellite sensors). Of the 37 different predictor variables we explored, only 22 were unique enough to warrant their inclusion when running our non-correlated model (see Table 2). Thus, although deriving the additional variables was not particularly laborious in the pixel-based assessments, it may not be warranted, at least in this study area, given the response across the breadth of classes.

In addition, it is worth noting that model parsimony is generally desirable. For instance, lower OOB-error estimates (i.e., better models) occur in RF classification with a lower number of variables (mtry) used for splitting the trees at each node and with a lower number of trees (ntree) constructed [74].

### 4.3. Object-Oriented Approaches for Wetland Classification

The use of the object-oriented approach allowed optimization of the classification results based on geometric and spectral homogeneity of image-objects. This, coupled with the RF classification approach, resulted in an overall classification of 84.6% and 90.4%, using the three and five most important predictor variables, respectively (see Tables 8 and 9). It is possible that further increased overall accuracy may be possible with the coupled OBIA and random forest plus additional predictors. However, as mentioned our accuracy achieved using five predictors (~90%) means only limited improvements can come from increasing the number of predictors. The OBIA approach requires substantial user inputs and processing; it is too time-consuming to consider the OBIA across the breadth of metrics (37) considered in this analysis.

However, OBIA may be useful for analyses, depending on characteristics of the “objects” within the study area. Thus, it is possible to harness the power of OBIA through iterative approaches of using different study-area segmentation parameters and scales to better generate image-object identification for subsequent input as predictor variables. For instance, in our study area, we found that a segmentation scale of 5 provided the greatest number of objects and was also the most informative; others may find a different scale useful [31,75,76]. Similar to this study’s exploration of segmentation scale on overall accuracy (see Table 8), Stumpf and Kerle [28] found decreasing model performance at larger segmentation scales. However, the smaller the segmentation scale, the more computational resources are required for data processing time and data storage for intermediate geospatial products. This factors into end-user decisions when processing high-resolution multispectral remote sensing data, particularly when additional derived and auxiliary dataset that are relevant for better wetland detection and delineation are integrated.

We therefore followed others (e.g., [28,29,31,32]) and first separated the image into segments and then used the RF classification algorithm to assign wetland classes based on the spectral data of the homogenous image-objects. We found that combining both OBIA and RF for segmentation and classification purposes, respectively, allowed the utilization of the strengths of each approach for better wetland delineation and mapping. Based on the results from our study, the hybrid OBIA-RF approach can likely be applied in similar settings where spectral information has more influence than shape in wetland class distinction and should be further explored, especially as processing power continues to increase and non-proprietary software packages and algorithms are becoming more available.

## 5. Conclusions

The use of remote sensing to classify wetland landscapes is critical to the understanding of the structure that begets wetland functions, especially as the world’s finite wetland resources are lost [77]. Field-based data plus robust spectral analyses from satellite platforms provide useful information for effective management. There are many approaches that can be used to classify the landscape with remotely sensed data, from established and relatively simplistic (e.g., ISODATA) to the novel and increasingly complex (e.g., random forest, neural networks, etc.). Similarly, many different band combinations creating spectral metrics are used to further discriminate amongst landscape features (e.g., NDVI). To facilitate subsequent remotely sensed wetland analyses, we conducted both pixel- and object-based analyses using parametric and non-parametric approaches and progressively incorporated spatial and spectral metrics. The pixel-based maximum likelihood and random forest approaches performed well using a parsimonious Quickbird band 3, WRI, and mean texture variables, with accuracies of 83.9% and 87.9%, respectively. The inclusion of an additional two predictors to our “parsimonious” model degraded our accuracy using pixel-based maximum likelihood and random forest approaches. However, when an object-based image analysis approach was coupled with the random forest analysis, our overall accuracy increased from 84.6% with three predictors to 89.6% with five predictors; more predictors allowed greater discrimination between the >5 million objects in the analysis. Segmentation scale had an effect on these results; larger objects resulted in smaller overall accuracies. Fewer objects did, however, decrease the processing and time requirements for the study—these trade-offs are thus important to consider when analyzing wetland-dominated landscapes. In addition, the object-oriented approach requires repeated iterations and additional subjective parameterization (e.g., compactness and smoothness values). Furthermore, OBIA was conducted using commercial software, an expense that these results suggest might not be warranted (as we found no significant difference between the overall accuracies in our analyses). Thus, we conclude that the random forest algorithm warrants its increased use as an analytical approach in effectively assessing and mapping wetland resources, be it through either pixel- or object-based approaches.

## Figures and Tables

**Table 1:** Description of the input predictor variables used in this study (B1–B4 are Quickbird multispectral bands, while B5–B37 are identifications assigned to the derived spatial and spectral matrices).

Input Data Layer (Stack)	Description	Equations
B1234	Quickbird multispectral bands	-
ARVI (B5)	Atmospherically resistant vegetation index [37,53]	$\frac{\left(\rho_{\text{NIR}}-\rho_{\text{RB}}\right)}{\left(\rho_{\text{NIR}}-\rho_{\text{RB}}\right)}$
BNDVI (B6)	Blue-normalized difference vegetation index [52]	$\frac{\left(\rho_{B}-\rho_{\text{NIR}}\right)}{\left(\rho_{B}+\rho_{\text{NIR}}\right)}$
DVI (B7)	Difference vegetation index [36,51]	*ρ_NIR_* − *ρ_R_*
GNDVI (B8)	Green-normalized difference vegetation index [52]	$\frac{\left(\rho_{\text{NIR}}-\rho_{G}\right)}{\left(\rho_{\text{NIR}}+\rho_{G}\right)}$
IPVI (B9)	Infrared percentage vegetation index [39]	$\frac{\rho_{\text{NIR}}}{\left(\rho_{\text{NIR}}+\rho_{R}\right)}$
NDVI (B10)	Normalized difference vegetation index [51]	$\frac{\left(\rho_{\text{NIR}}-\rho_{R}\right)}{\left(\rho_{\text{NIR}}+\rho_{R}\right)}$
NDWI (B11)	Normalized difference water index [54]	$\frac{\left(\rho_{G}-\rho_{\text{NIR}}\right)}{\left(\rho_{G}+\rho_{\text{NIR}}\right)}$
SAVI (B12)	Soil adjusted vegetation index [38]	$\frac{\left(\rho_{\text{NIR}}-\rho_{R}\right)}{\left(\rho_{\text{NIR}}+\rho_{R}+L\right)}\times (1+L)$
WRI (B13)	Water ratio index [55]	$\left(\frac{\rho_{G}+\rho_{R}+\rho_{\text{NIR}}}{\rho_{B}}\right)$
Ratio Transformation	Ratio of reflectance spectra [52]; B14–B19	*ρ_B_/ρ_G_; ρ_R_/ρ_B_; ρ_R_/ρ_G_; ρ_NIR_/ρ_B_; ρ_NIR_/ρ_G_; ρ_NIR_/ρ_R_*
Texture Metrics	Texture variables (contrast (B20), correlation (B21), dissimilarity (B22), entropy (B23), homogeneity (B24), mean (B25), 2nd moment (B26), and variance (B27)) computed as a measure of Gray Level Co-occurrence Matrix (GLCM) using Band4 (Harris Geospatial Solutions, Herndon, VA, USA, version 5.3).	Source: Harris Geospatial, Texture Metrics Background. Available online: www.harrisgeospatial.com/docs/backgroundtexturemetrics.html (accessed on 26 December 2017).
Topography Metrics	Advanced Spaceborne Thermal Emission and Reflection Radiometer (ASTERGDEM)-Global Digital Elevation Model (GDEM). GDEM-derived variables (aspect (B28), cross-sectional convexity (B29), DEM (B30), longitudinal convexity (B31), maximum curvature (B32), minimum curvature (B33), plan convexity (B34), profile convexity (B35), RMS error (B36), and slope (%; B37) (Harris Geospatial Solutions, Herndon, VA, USA, version 5.3)).	Source: Harris Geospatial, Topographic Modeling Background. Available online: www.harrisgeospatial.com/docs/backgroundtopographicmodeling.html(accessed on 26 December 2017).

**Table 2:** Correlation matrix of predictor variables. Linear correlations (|r| ≥ 0.89) are shaded grey. Quickbird bands 1–4 are designed B1–4; see text for additional abbreviations.

Predictor	B1	B2	B3	B4	B5	B6	B7	B8	B9	B10	B11	B12	B13	B14	B15	B16	B17	B18	B19	B20	B21	B22	B23	B24	B25	B26	B27	B28	B29	B30	B31	B32	B33	B34	B35	B36	B37
**B1**	1.00																																				
**B2**	**0.96**	1.00																																			
**B3**	**0.96**	**0.96**	1.00																																		
**B4**	0.62	0.74	0.62	1.00																																	
**B5**	−0.08	−0.08	−0.08	−0.02	1.00																																
**B6**	0.55	0.66	0.56	**0.94**	−0.01	1.00																															
**B7**	−0.16	−0.15	−0.13	−0.05	−0.02	−0.05	1.00																														
**B8**	0.5	0.6	0.49	**0.93**	0	**1**	−0.03	1.00																													
**B9**	0.28	0.4	0.26	0.85	0.02	**0.94**	0	**0.96**	1.00																												
**B10**	0.28	0.4	0.26	0.85	0.02	**0.94**	0	**0.96**	**1**	1.00																											
**B11**	−0.50	0.31	−0.49	−0.93	0	**−1.00**	0.03	**−1.00**	**−0.96**	**−0.96**	1.00																										
**B12**	0.28	0.4	0.26	0.85	0.02	**0.94**	0	**0.96**	**1**	**1**	**−0.96**	1.00																									
**B13**	0.62	0.75	0.63	**0.99**	−0.02	**0.95**	−0.05	**0.94**	0.85	0.85	**−0.94**	0.85	1.00																								
**B14**	−0.68	−0.86	−0.74	−0.82	0.05	−0.77	0.11	−0.71	−059	−0.59	0.71	−059	−0.85	1.00																							
**B15**	**0.93**	**0.94**	**0.99**	0.65	−0.07	0.62	−0.12	0.56	0.32	0.32	−0.56	0.32	0.68	−0.77	1.00																						
**B16**	**0.9**	0.84	**0.95**	0.51	−0.06	0.51	−0.10	0.46	0.2	0.2	−0.46	0.2	0.53	−0.59	**0.96**	1.00																					
**B17**	0.46	0.6	0.46	**0.98**	0	**0.95**	−0.02	**0.95**	**0.91**	**0.91**	**−0.95**	**0.91**	**0.98**	−0.75	0.51	0.37	1.00																				
**B18**	0.41	0.53	0.4	**0.95**	0.01	**0.95**	−0.01	**0.96**	**0.93**	**0.93**	**−0.96**	**0.93**	**0.95**	−0.68	0.46	0.33	**0.99**	1.00																			
**B19**	0.17	0.31	0.14	0.85	0.03	0.85	0.02	**0.88**	**0.93**	**0.93**	−0.88	**0.93**	0.84	−0.54	0.2	0.06	**0.94**	**0.96**	1.00																		
**B20**	0.16	0.2	0.17	0.28	0.01	0.25	0.03	0.25	0.24	0.24	−0.25	0.24	0.27	−0.23	0.17	0.12	0.27	0.25	0.23	1.00																	
**B21**	−0.17	−0.16	−0.17	−0.18	0	−0.28	−0.01	−0.28	−0.26	−0.26	0.28	−0.26	−0.19	0.14	−0.19	−0.20	−0.17	−0.19	−0.14	−0.16	1.00																
**B22**	0.21	0.26	0.22	0.38	0.01	0.42	0.03	0.42	0.41	0.41	−0.42	0.41	0.38	−0.31	0.24	0.19	0.38	0.38	0.35	0.87	−0.38	1.00															
**B23**	0.33	0.36	0.35	0.45	0	0.6	−0.04	0.59	0.56	0.56	−0.59	0.56	0.47	−0.38	0.39	0.38	0.45	0.47	0.39	0.36	−0.54	0.69	1.00														
**B24**	−0.24	−0.27	−0.25	−0.40	−0.01	−0.51	−0.02	−0.52	−050	−0.50	0.52	−050	−0.41	0.32	−0.29	−0.26	−0.41	−0.42	−0.37	−0.53	0.55	−0.87	−0.89	1.00													
**B25**	0.63	0.74	0.62	**1**	−0.02	**0.94**	−0.05	**0.93**	0.85	0.85	**−0.93**	0.85	0.99	−0.82	0.65	0.51	**0.98**	**0.95**	0.85	0.28	−0.18	0.38	0.46	−0.40	1.00												
**B26**	−0.34	−0.36	−0.35	−0.42	0.01	−0.57	0.04	−0.56	−052	−0.52	0.56	−052	−0.44	0.36	−0.40	−0.39	−0.41	−0.44	−0.34	−0.30	0.6	−0.62	−0.98	0.85	−0.43	1.00											
**B27**	0.15	0.2	0.15	0.28	0.01	0.27	0.02	0.27	0.26	0.26	−0.27	0.26	0.28	−0.24	0.16	0.11	0.28	0.26	0.24	0.79	−0.09	0.75	0.37	−0.50	0.28	−0.30	1.00										
**B28**	−0.01	0	−0.01	0	−0.01	0.02	−0.01	0.02	0.02	0.02	−0.02	0.02	0.01	−0.01	0	0	0.01	0.01	0.01	0.01	−0.07	0.04	0.04	−0.05	0	−0.05	0	1.00									
**B29**	−0.01	−0.01	−0.01	0.01	0	0.01	0	0.02	0.02	0.02	−0.02	0.02	0.01	0	−0.01	−0.01	0.02	0.02	0.03	0.02	−0.02	0.02	0.02	−0.02	0.01	−0.02	0.02	−0.01	1.00								
**B30**	0.18	0.19	0.19	0.15	−0.02	0.14	−0.07	0.13	0.08	0.08	−0.13	0.08	0.16	−0.16	0.19	0.17	0.13	0.12	0.08	0.13	−0.09	0.15	0.14	−0.13	0.15	−0.14	0.09	0.03	0.02	1.00							
**B31**	0	0.01	0.01	−0.01	0.01	0	0	0	0	0	0	0	0	−0.01	0.01	0.01	−0.01	−0.01	−0.01	0.02	0	0.01	0	0	0	−0.01	0.02	0.02	0.08	0.09	1.00						
**B32**	−0.01	−0.01	0	0.01	0.02	0.01	0.02	0.01	0.01	0.01	−0.01	0.01	0.01	0	0	0	0.01	0.01	0.02	0	−0.03	0	0.01	−0.01	0.01	−0.02	0	0.12	0.19	0.08	0.73	1.00					
**B33**	0.02	0.01	0.02	−0.01	0	0	−0.02	−0.01	−0.01	−0.01	0.01	−0.01	−0.01	−0.01	0.01	0.01	−0.02	−0.02	−0.02	0.03	0.02	0.02	0	0	−0.01	0	0.03	−0.09	0.19	0.05	0.72	0.09	1.00				
**B34**	−0.01	0	0	−0.01	0	−0.01	0	−0.01	−0.01	−0.01	0.01	−0.01	−0.01	−0.01	0	0	−0.01	−0.01	−0.01	−0.02	0.01	−0.02	−0.02	0.01	−0.01	0.01	−0.02	0.01	−0.83	−0.02	−0.07	−0.15	−0.17	1.00			
**B35**	0	0	0.01	−0.01	0.02	0	0	0	−0.01	−0.01	0	−0.01	0	−0.01	0.01	0.01	−0.01	−0.01	−0.01	0.01	0.03	0	0	0.01	−0.01	0.01	0.01	0	0.06	0.06	0.77	0.57	0.54	−0.07	1.00		
**B36**	0.01	0.01	0.01	0.04	0.01	0.04	−0.02	0.04	0.04	0.04	−0.04	0.04	0.03	−0.02	0.01	0.01	0.04	0.04	0.04	0.01	0	0.02	0.03	−0.03	0.04	−0.02	0.02	−0.01	0.06	−0.01	−0.02	0.19	−0.20	0.06	0.01	1.00	
**B37**	−0.02	−0.02	−0.02	0.02	0.01	0.01	0.03	0.02	0.02	0.02	−0.02	0.02	0.01	0.01	−0.01	−0.02	0.02	0.03	0.03	−0.03	−0.03	−0.01	0.01	−0.01	0.02	−0.02	−0.02	0.15	0	0.02	0.02	0.67	−0.66	0.02	0.03	0.27	1.00

B1 = Quickbird band 1, B2 = Quickbird band 2, B3 = Quickbird band 3, B4 = Quickbird band 4, B5 = ARVI, B6 = BNDVI, B7 = DVI, B8 = GNDVI, B9 = IPVI, B10 = NDVI, B11 = NDWI, B12 = SAVI, B13 = WRI, B14 = B1/B2, B15 = B3/B1, B16 = B3/B2, B17 = B4/B1, B18 = B4/B2, B19 = B4/B3, B20 = Texture (Contrast), B21 = Texture (Correlation), B22 = Texture (Dissimilarity), B23 = Texture (Entropy), B24 = Texture (Homogeneity), B25 = Texture (Mean), B26 = Texture (2nd moment), B27 = Texture (Variance), B28 = Aspect, B29 = Cross-sectional convexity, B30 = DEM, B31 = Longitudinal convexity, B32 = Maximum curvature, B33 = Minimum curvature, B34 = Plain convexity, B35 = Profile convexity, B36 = RMS error, B37 = Slope (%).

**Table 3:** McNemar’s chi-squared test summary of the classification accuracy differences observed by the three classifiers. No significant differences were found between the three classification methods we employed when using either a three- or five-layer stack.

Classifier	Chi-Squared		*p*-Value	

	Pixel-Based ML	Object-Based RF	Pixel-Based ML	Object-Based RF
		**Three-Layer Stack**		

**Pixel-based RF**	0.083	0.078	0.774	0.780
**Pixel-based ML**	-	0.100	-	0.752

		**Five-Layer Stack**		

**Pixel-based RF**	0.128	0.058	0.720	0.810
**Pixel-based ML**	-	0.096	-	0.756

**Table 4:** Pixel-based random forest classification accuracy on training and testing datasets with various input variable combinations (including 95% confidence interval, CI). The data are sorted based on overall accuracy of the testing data.

Predictor Variables	Training Data OOB Error (%)	Testing Data		
		Overall Accuracy		
		%	95%	CI
All non-correlated variables (with WRI, not mean texture; 22 variables)	1.3	72.7	72.6	72.9
All non-correlated variables (with mean texture, not WRI; 22 variables)	0.3	80.1	80.0	80.2
All non-correlated variables including both WRI and mean texture (23 variables)	0.4	80.1	80.1	80.2
All (37) variables	0.8	84.6	84.5	84.7
Ten most important variables	1.0	84.6	84.5	84.6
Five most important variables	0.9	84.9	84.8	85.0
Fifteen most important variables	1.2	85.6	85.5	85.8
Most parsimonious model (three variables: B3, WRI, and mean texture)	1.4	87.9	87.8	88.0

**Table 5:** Pixel-based random forest classification class confusion matrix (pixel-counts) for genus-level wetland classes and aquatic habitats (three-layer stack). PA, UA, and OA are producer’s, user’s, and overall accuracy, respectively. See the legend in Figure 5a for additional information regarding the wetland class community composition.

Wetland Class	1	2	3	4	5	6	7	8	9	10	11	12	13	14	15	16	17	18
1.00	55	1.00	0	0	0	0	0	0	0	0	0	0	0	0	0	0	0	0
2	0	58	0	0	0	0	0	0	0	0	0	0	0	0	0	0	0	0
3	5	0	21	0	0	0	9	0	0	0	0	0	0	0	0	0	0	0
4	0	0	30	68	0	0	0	0	0	0	0	0	0	0	0	0	0	0
5	0	0	12	0	63	19	0	0	0	0	0	0	0	0	0	0	0	0
6	0	0	0	0	0	40	0	0	0	0	0	0	0	0	0	0	0	0
7	1.00	0	0	0	0	0	51	0	9	6	0	0	0	0	0	0	0	0
8	0	0	0	0	0	2	0	58	0	5	0	0	0	0	0	0	0	0
9	0	0	0	0	0	0	0	0	53	0	0	0	0	15	0	0	0	0
10	0	0	0	0	0	0	0	2	0	51	0	0	0	0	0	0	0	0
11	0	0	0	0	0	0	0	0	1.00	0	62	0	0	0	0	0	0	0
12	0	0	0	0	0	0	0	0	0	0	0	61	0	0	0	0	0	0
13	0	0	0	0	0	0	0	0	0	0	0	0	84	0	0	0	0	0
14	0	0	0	0	0	0	0	0	0	0	2	0	0	40	0	0	0	0
15	0	0	0	0	0	0	0	0	0	0	0	0	0	0	65	0	2	0
16	0	0	0	0	0	0	0	0	0	0	0	3	0	7	0	44	3	0
17	0	0	0	0	0	0	0	0	0	0	0	0	0	0	0	0	53	0
18	0	0	0	0	0	0	0	0	0	0	0	0	0	0	0	0	1	56

PA (%)	90.2	98.1	33.1	100	100	65.5	84.3	97.0	84.2	82.9	96.9	95.3	100	64.4	100	100	90.0	100
UA (%)	97.2	100	59.7	69.3	67	100	76.8	89.3	78.0	96.7	98.4	100	100	95.2	97.0	77.1	100	98.3
OA (%)	87.9																	

**Table 6:** The importance of each predictor variable (based on MDG) for both pixel-based RF classification approaches (cumulative count of importance out of 100 iterations; only the top 20 important variables are included). Abbreviations are found within the text and Table 1.

Variable Importance Rank															
Predictor Variable	1st	2nd	3rd	4th	5th	6th	7th	8th	9th	10th	11th	12th	13th	14th	15th
Quickbird B1	-	-	-	-	-	-	-	1.00	5	12	30	45	5	2	-
Quickbird B2	-	-	-	-	100	-	-	-	-	-	-	-	-	-	-
Quickbird B3	-	-	1.00	99	-	-	-	-	-	-	-	-	-	-	-
Quickbird B4	-	100	-	-	-	-	-	-	-	-	-	-	-	-	-
ARVI	-	-	-	-	-	-	-	-	-	-	-	-	-	-	-
B1/B2	-	-	-	-	-	-	-	-	-	-	-	-	-	-	-
BNDVI	-	-	-	-	-	51	49	-	-	-	-	-	-	-	-
DVI	-	-	-	-	-	-	-	57	18	19	3	3	-	-	-
GNDVI	-	-	-	-	-	-	-	12	24	25	25	14	-	-	-
IPVI	-	-	-	-	-	-	-	-	-	-	-	1.00	10	18	20
NDVI	-	-	-	-	-	-	-	-	-	-	-	-	5	20	22
B4/B1	-	-	-	-	-	49	51	-	-	-	-	-	-	-	-
B4/B2	-	-	-	-	-	-	-	14	25	28	15	17	1.00	-	-
B4/B3	-	-	-	-	-	-	-	-	-	-	-	-	6	24	19
B3/B1	-	-	-	-	-	-	-	-	-	-	-	8	68	19	4
B3/B2	-	-	-	-	-	-	-	-	-	-	-	-	-	-	-
SAVI	-	-	-	-	-	-	-	-	-	-	-	-	4	17	35
WRI	-	-	99	1.00	-	-	-	-	-	-	-	-	-	-	-
Mean texture	100	-	-	-	-	-	-	-	-	-	-	-	-	-	-
NDWI	-	-	-	-	-	1	-	16	28	16	27	12	1	-	-

**Table 7:** Pixel-based ML classification class confusion matrix (pixel-counts) for genus-level wetland classes and aquatic habitats (three-layer stack). PA, UA, and OA are producer’s, user’s, and overall accuracy, respectively. See the legend in Figure 5a for additional information regarding the wetland class community composition.

Wetland Class	1	2	3	4	5	6	7	8	9	10	11	12	13	14	15	16	17	18
1.00	51	0	0	0	0	0	0	0	0	0	0	0	0	0	0	0	0	0
2	0	55	0	0	0	0	0	0	0	0	0	0	0	0	0	0	0	0
3	9	4	44	0	20	10	0	0	0	0	0	0	0	0	0	0	0	0
4	0	0	17	68	0	0	0	0	0	0	0	0	0	0	0	0	0	0
5	0	0	2	0	42	0	0	0	0	0	0	0	0	0	0	0	0	0
6	0	0	0	0	1.00	29	0	0	0	0	0	0	0	0	0	0	0	0
7	1.00	0	0	0	0	21	61	0	20	1.00	0	0	0	0	0	0	0	0
8	0	0	0	0	0	1.00	0	54	0	0	0	0	0	0	0	0	0	0
9	0	0	0	0	0	0	0	0	24	0	1.00	0	0	27	0	0	0	0
10	0	0	0	0	0	0	0	6	0	61	0	0	0	0	0	0	0	0
11	0	0	0	0	0	0	0	0	19	0	62	0	0	0	0	0	0	0
12	0	0	0	0	0	0	0	0	0	0	0	58	2	0	0	0	0	0
13	0	0	0	0	0	0	0	0	0	0	0	6	82	0	0	0	3	0
14	0	0	0	0	0	0	0	0	0	0	1.00	0	0	32	4	0	0	0
15	0	0	0	0	0	0	0	0	0	0	0	0	0	0	61	0	0	0
16	0	0	0	0	0	0	0	0	0	0	0	0	0	3	0	44	0	0
17	0	0	0	0	0	0	0	0	0	0	0	0	0	0	0	0	55	0
18	0	0	0	0	0	0	0	0	0	0	0	0	0	0	0	0	1	56

PA (%)	83.6	93.2	69.8	100.0	66.7	47.5	100.0	90.0	38.1	98.4	96.9	90.6	97.6	51.6	93.8	100.0	93.2	100.0
UA (%)	100.0	100.0	50.6	80.0	95.5	96.7	58.7	98.2	46.2	91.0	76.5	96.7	90.1	86.5	100.0	93.6	100.0	98.2
OA (%)	83.9																	

**Table 8:** Object-based random forest classification accuracy on training and testing datasets with various input variable combinations. Quickbird bands 2, 3, and 4 are represented as B2, B3, and B4, respectively; also included are WRI (Water Ratio Index) and mean texture.

Predictor Variables	Training Data OOB Error (%)	Testing Data		
		Overall Accuracy		
		%	95%	CI
**B3 + WRI + mean texture**				

Scale 5	0.4	84.6	84.3	84.8
Scale 10	0.2	67.7	67.4	67.9
Scale 15	1.9	67.6	67.5	67.6
Scale 30	2.3	46.7	46.2	47.1
Scale 50	6.0	57.6	57.1	58.2
Scale 100	22.4	37.4	36.9	37.9

**Scale 5**	0.3	90.4	90.3	90.4
**B2 + B3 + B4 + WRI + mean texture**				

**Table 9:** Object-based random forest classification (segmentation scale of 5) confusion matrix (pixel-counts) for genus-level wetland classes and aquatic habitats (three-layer stack). PA, UA, and OA are producer’s, user’s, and overall accuracy, respectively. See the legend in Figure 5a for additional information regarding the wetland class community composition.

Wetland Class	1	2	3	4	5	6	7	8	9	10	11	12	13	14	15	16	17	18
1.00	60	0	0	0	0	0	0	0	0	0	0	0	0	0	0	0	0	0
2	0	59	0	0	0	0	0	0	0	0	0	0	0	0	0	0	0	0
3	1.00	0	37	0	0	0	33	0	0	0	0	0	0	0	0	0	0	0
4	0	0	25	67	0	0	0	0	0	0	0	0	0	0	0	0	0	0
5	0	0	0	1.00	63	0	0	0	0	0	0	0	0	0	0	0	0	0
6	0	0	1.00	0	0	46	0	5	0	0	0	0	0	0	0	0	0	0
7	0	0	0	0	0	0	28	0	0	0	0	0	0	0	0	0	0	0
8	0	0	0	0	0	15	0	55	0	36	0	0	0	0	0	0	0	0
9	0	0	0	0	0	0	0	0	62	0	0	0	0	21	0	0	0	0
10	0	0	0	0	0	0	0	0	0	26	3	0	0	0	0	0	0	0
11	0	0	0	0	0	0	0	0	0	0	59	0	0	0	0	0	0	0
12	0	0	0	0	0	0	0	0	0	0	0	63	0	0	0	0	0	0
13	0	0	0	0	0	0	0	0	0	0	0	0	84	0	0	4	0	0
14	0	0	0	0	0	0	0	0	1.00	0	2	0	0	41	0	0	0	0
15	0	0	0	0	0	0	0	0	0	0	0	0	0	0	65	0	13	0
16	0	0	0	0	0	0	0	0	0	0	0	1.00	0	0	0	40	0	0
17	0	0	0	0	0	0	0	0	0	0	0	0	0	0	0	0	36	0
18	0	0	0	0	0	0	0	0	0	0	0	0	0	0	0	0	11	56

PA (%)	98.4	100.0	58.4	98.5	100.0	75.4	45.9	91.7	98.4	41.9	92.2	98.4	100.0	66.1	100.0	90.9	60.4	100.0
UA (%)	100.0	100.0	52.0	72.7	98.4	88.5	100.0	52.0	74.7	89.7	99.7	100.0	95.5	93.2	83.8	97.6	100.0	83.9
OA (%)	84.6																	
